# Sleep facilitates spatial memory but not navigation using the Minecraft Memory and Navigation task

**DOI:** 10.1073/pnas.2202394119

**Published:** 2022-10-17

**Authors:** Katharine C. Simon, Gregory D. Clemenson, Jing Zhang, Negin Sattari, Alessandra E. Shuster, Brandon Clayton, Elisabet Alzueta, Teji Dulai, Massimiliano de Zambotti, Craig Stark, Fiona C. Baker, Sara C. Mednick

**Affiliations:** ^a^Department of Cognitive Sciences, School of Social Sciences, University of California, Irvine, CA 92697;; ^b^Department of Neurobiology and Behavior, School of Biological Sciences, University of California, Irvine, CA 92697;; ^c^Center for Health Sciences, SRI International, Menlo Park, CA 94025;; ^d^Brain Function Research Group, School of Physiology, University of the Witwatersrand, Johannesburg, 2193, South Africa

**Keywords:** sleep, spatial memory, spatial navigation

## Abstract

Sleep facilitates hippocampal-dependent memories, supporting the acquisition and maintenance of internal representation of spatial relations within an environment. In humans, however, findings have been mixed regarding sleep’s contribution to spatial memory and navigation, which may be due to task designs or outcome measurements. We developed the Minecraft Memory and Navigation (MMN) task for the purpose of disentangling how spatial memory accuracy and navigation change over time, and to study sleep’s independent contributions to each. In the MMN task, participants learned the locations of objects through free exploration of an open field computerized environment. At test, they were teleported to random positions around the environment and required to navigate to the remembered location of each object. In study 1, we developed and validated four unique MMN environments with the goal of equating baseline learning and immediate test performance. A total of 86 participants were administered the training phases and immediate test. Participants’ baseline performance was equivalent across all four environments, supporting the use of the MMN task. In study 2, 29 participants were trained, tested immediately, and again 12 h later after a period of sleep or wake. We found that the metric accuracy of object locations, i.e., spatial memory, was maintained over a night of sleep, while after wake, metric accuracy declined. In contrast, spatial navigation improved over both sleep and wake delays. Our findings support the role of sleep in retaining the precise spatial relationships within a cognitive map; however, they do not support a specific role of sleep in navigation.

## Significance

Sleep facilitates hippocampal-dependent memories, and the hippocampus uniquely supports the spatial acquisition and representation of environments. However, sleep’s contribution to how specific locations within environments (spatial memory) are retained and the movements to them (navigation) have had mixed findings, possibly due to task designs, familiarity of environments, or measurements. Additionally, prior research has not been able to disentangle the contributions of sleep to spatial memory from navigation. To meet this need, we developed the Minecraft Memory and Navigation (MMN) task to study how spatial memory and navigation changes over time and during sleep. Our findings support the role of sleep in retaining precise spatial relationships within a cognitive map. However, they do not support the role of sleep in navigation.

Sleep has been repeatedly demonstrated to be the keystone in facilitating the consolidation of long-term memories. These sleep-dependent processes appear to uniquely support the stabilization of hippocampal-dependent information, specifically the interconnected details of episodic events that can be consciously and explicitly recalled (1, 2). Episodic details are typically embedded within the nexus of spatial contexts (3, 4), and the hippocampus has been shown to uniquely support the acquisition and internal representation of spatial environments, termed cognitive maps (5, 6). Cognitive maps are built through environment exploration, and hippocampal place cells respond to location-specific positions (6). In subsequent sleep periods, after task learning, place cells have been found to refire in sequence, known as replay (7–9). Though replay of place cell firing is a prominent hypothesized mechanism for sleep-dependent hippocampal memory consolidation (10–13), few studies have shown a direct relationship between sleep-based hippocampal replay and subsequent spatial memory improvement (14, 15).

Navigation benefits the development of cognitive maps via the linking of associated spatial relationships between the self, objects, landmarks, and environment boundaries (5, 6, 16, 17). Spatial relationships can be represented as allocentric, or perspective independent, in which the world relationships remain stable regardless of one’s self movement. Cognitive maps are inherently allocentric, as they are an internal representations of the spatial relationships within a given environment regardless of the position of the self (5, 6). Primary neural areas associated with allocentric representations include the medial temporal lobe, specifically the hippocampus and entorhinal cortex, with secondary areas suggested to be the perirhinal cortex, amygdala, and fusiform gyrus (18, 19). Alternatively, spatial relationships can be egocentric and referenced to the self. This relies on a constant updating of environmental spatial relationships in accordance with one’s own movements and is associated with the posterior parietal cortex (6, 20, 21). Navigation as a behavior is a complex multisensory process that integrates memory, perception, vestibular signals, the motor system, and decision making, thus, requiring the facilitation of a wide range of neural structures, of which the hippocampus is only one (5, 21–24). Navigation can also be influenced by a number of factors, including age, sex, brain injury, and personality (16, 23–27) and can be accomplished with allocentric strategies that use metric configurational knowledge of the environment or egocentric strategies, that involve response-base strategies, also known as stimulus–response strategies, which use proximal cues as they relate to oneself during navigation. How sleep supports navigational behavior remains an open question in humans.

Behaviorally, spatial memory and the navigation system are fundamentally intertwined, and disentangling the contributions of sleep to spatial memory and spatial navigation independently remains difficult. Prior attempts to understand the impact of sleep on the formation of long-term spatial memory resulted in mixed findings, with only some studies demonstrating a sleep-dependent performance benefit (28–33) while others have not (34, 35). This lack of consistent findings is in line with other spatial memory and navigation research and may be the result of differences in task design, familiarity of the learned environments, or that participants can employ either egocentric or allocentric navigation strategies to complete the same task (21, 22, 36). Most of the sleep-dependent tasks have had similar environment designs, such as navigating a novel route between a start and end location, whether it be landmark, object, or start/end of a maze. Among different existing performance measures, the participants’ spatial memory is assessed as a change in performance in the total amount of time taken, the number of direction changes, the distance away from an end target location, or the total path length from a starting point to a target location (22, 32). These navigation outcome metrics do not easily provide the opportunity for participants to demonstrate their metrically precise spatial memories separate from their navigation behaviors. Thus, while some studies have supported the existence of sleep-dependent spatial consolidation, they have not been able to tease apart the potential contributions of sleep to navigation behaviors versus spatial memories.

Early studies evaluated experience-dependent changes to sleep due to spatial learning, without comparing to a wake control group. Meier-Koll et al. found that participants who learned a city-based virtual maze, navigating from specific start to end points, had an increased total amount of stage 2 sleep and increased count of spindles after encoding (37). Similarly, Peigneux et al. trained participants on a virtual town and tested them by measuring the distance between participants’ position at the end of a timed test trial and the actual end target location, with greater distance suggesting worse memory (38). They showed that the hippocampus was activated, as assessed with positron emission tomography (PET), during the navigation of a virtual town, and was similarly activated during slow wave sleep following learning the task. This neural activity correlated with next day behavioral performance improvement. Subsequent studies tested for a specialized role of sleep in spatial memory consolidation by administering Peigneux’s virtual navigation task and having participants either sleep or undergo sleep deprivation for the night after learning (30, 39). Subsequent testing found no behavioral performance differences between the two conditions; however, participants who slept showed functional reorganization of the spatial memory with increased striatal activity after sleep that correlated with behavioral performance (30, 39). Javadi et al. used a similar place-finding virtual navigation task but attempted to strengthen the sleep-dependent aspect by training participants to associate locations with specific value-laden objects (40). Using this model, they, too, found no specific benefit of sleep on memory accuracy when identifying the spatial relations between objects; however, their sleep group showed value generalization for high value objects as a function of spatial proximity.

In contrast, other studies have found sleep-specific spatial memory benefits (31–35). Ferrara et al. were one of the first to show that sleep supported the consolidation of virtual maze representations such that with sleep compared to extended wake, participants were faster at navigating routes between two learned landmarks (31). Following this, Nguyen et al. found that after sleep, compared to wake, navigation accuracy improved as measured by a decrease in the total path length, fewer route changes (i.e., backtracking), and reduced total completion time (32). As age is thought to impact spatial memory and navigation, Varga et al. used Nguyen et al.’s virtual task to investigate sleep-dependent spatial performance differences between younger and older adults (41). Younger, but not older participants, showed sleep-dependent performance improvements, as measured by faster completion time, which also correlated with frontal slow wave activity. More recently, Noack et al. showed that sleep preferentially supported route knowledge and insight into the explicit regionalized map structure (33). Following this study, Noack et al. showed that sleep also facilitated the use of flexible spatial strategies to switch between landmark and boundary references when placing objects in previously learned locations (34). However, none of these studies have investigated the longitudinal impact of sleep independently and interactionally on spatial memory and navigation.

Based on the need to disentangle the contributions of sleep to spatial memory and spatial navigation independently and understand changes in these processes over time, we developed the Minecraft Memory and Navigation task (MMN). The MMN is an open field task, that can be remotely administered, in which participants learn the locations of objects during free exploration and are tested on their spatial memory while recording each step-by-step movement. Following on these ideas, our task has the ability to disentangle the two spatial processes by providing knowledge of the metric precision of the underlying spatial relationships while also monitoring participants’ total path length, orientation, and relative proximity to the target locations. To achieve our goals of investigating the independent contributions of sleep to spatial memory and spatial navigation and create a task with longitudinal potential, we performed two independent studies. In study 1, we created and evaluated training and immediate test performance on four distinct environments with the goal of validating a task with longitudinal spatial memory and navigation measurement capabilities. In study 2, participants were trained on an environment and tested immediately and 12 h later after a period of sleep or wake. In line with prior studies, we hypothesized that sleep, compared to wake, would benefit spatial memory, specifically, the underlying metrically accurate locations of objects. Given the prior mixed sleep-dependent spatial cognition literature and that navigation is a dynamic process that integrates a host of cognitive processes, we did not have a specific hypothesis regarding a benefit of sleep on navigation.

## Results

### Study 1.

**Fig. 1.**
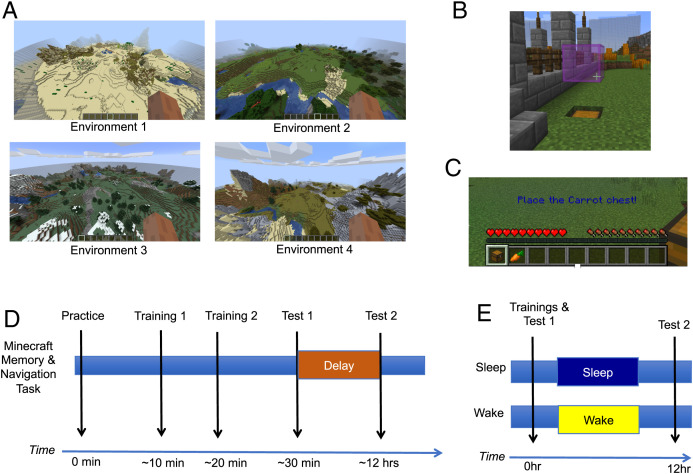
Minecraft Memory and Navigation task. (*A*) Bird’s eye view of all environments. Participants experienced environments in first person. (*B*) Pink flags marked the object locations during both free exploration trainings. (*C*) During testing. Objects were cued for recall with written and visually aided instructions. Hearts on the screen refer to Minecraft’s lives. All players played within a single life and these were irrelevant to the task. (*D*) MMN task timeline. Participants were provided task instructions and administered a practice environment. In the task, participants completed two consecutive free-exploration trainings, in which they were to find all 12 objects. After trainings, participants were administered an immediate cued recall test and then a second, delay test. Study 1 evaluated only trainings and test 1, performance. (*E*) Study 2 timeline. Participants completed the two consecutive free-exploration trainings and immediate test. Twelve hours later, after a night of sleep or day of wake, participants completed test 2.

We sought to validate the four MMN environments (see Figure 1 and described below in *Environment Design* section) for repeated remote spatial memory and navigation testing using a between-subject design. We evaluated training and immediate test (see Fig. 1*D* for timeline).

**Fig. 2.**
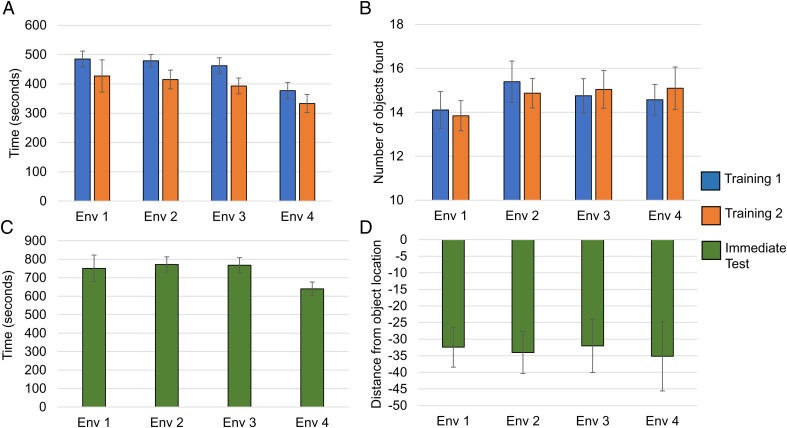
Minecraft environment training and test results. (*A*) Training time. There were no significant differences between training 1 and training 2 in the amount of time spent in the free-exploration phases. (*B*) Number of objects found. No significant differences between environments were found in the number of objects found across training sessions. (*C*) Test time. Participants spent equivalent time across the four environments in the testing phase. (*D*) Distance from object location. We found no differences between immediate spatial memory performance across environments.

To evaluate baseline learning in training across the four environments, we measured the number of objects found during a training phase and the amount of time spent within a training phase (Fig. 2 *A* and *B*). We evaluated the amount of time spent in trainings to find all the chests (Fig. 2*A*). We found a significant main effect of training [*F*(1, 82) = 11.096, *P* = 0.001, η^2^ = 0.119] with participants spending significantly greater time in training session 1 than training session 2 finding the objects. We found a significant main effect of environment [*F*(3, 82) = 2.872, *P* = 0.041], with participants assigned to environment 4 appearing to spend less time in training; however, none of the environmental comparisons withstood Bonferroni correction (alpha = 0.008). We also did not find an interaction between training time and environment [*F*(3, 82) = 0.129, *P* = 0.942, η^2^ = 0.005]. Across environments, when evaluating the number of objects found for each training, we did not find a main effect of environment [*F*(3, 82) = 0.648, *P* = 0.587, effect of training session [*F*(1, 82) = 0.02, *P* = 0.887, η^2^ = 0.0; Fig. 2*B*] or interaction between training and environment [*F*(3, 82) = 0.326, *P* = 0.806, η^2^ = 0.012]. Thus, the learning rate and exposure to chest locations was equivalent across the two free-exploration trainings regardless of environment assignment.

### Spatial Memory.

We evaluated spatial memory accuracy across the four environments at immediate test because equivalent outcomes from learning would further validate that our environments were commensurate (Fig. 2 *C* and *D*). Participants also spent a similar amount of time placing the chests irrespective of the environment [*F*(3, 85) = 1.316, *P* = 0.275; Fig. 2*C*). We also found no significant differences in the chest-location accuracy across the four environments [*F*(3, 84) = 0.064, *P* = 0.979, η^2^ = 0.002; Fig. 2*D*).

### Study 1 Interim Discussion.

Here we demonstrate success in developing our MMN task, in which participants learned the locations of unique objects within an open arena during free exploration and were subsequently tested using cued recall. Our results show that across our environments, participants had similar training experiences, as evidenced by similar amounts of time spent exploring the environments and that they opened an equivalent number of chests when attempting to find each unique object. At test, subjects spent equal amounts of time navigating to and placing objects and had equivalent rates of spatial location accuracy. Although one environment took a shorter time to explore, this difference was not significant and did not equate to a memory difference across environments. As such, our first study validates our four environments providing the opportunity to explore how spatial memory and navigation change over delays filled with sleep or wake.

### Study 2 Results.

**Fig. 3.**
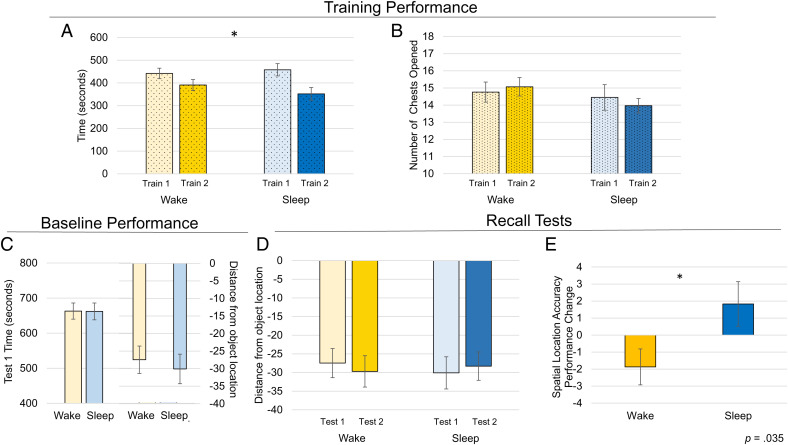
Minecraft sleep-dependent memory training session and test results. (*A*) Exploration time. Participants found and opened the 12 chests significantly faster in training 2 than in training 1. (*B*) Number of chests opened. No significant differences between conditions were found in the number of chests opened across trainings. Participants were required to open each chest at least once to identify and learn each unique object to proceed to the next phase of the task. Alternatively, there was a 10-min training session limit. (*C*) Baseline performance. *Left* side of graph: Participants spent the same amount of time in test 1 regardless of condition. *Right* side of graph: Participants placed the objects at equivalent distances in test 1, regardless of condition. (*D*) Distance from location. We found a significant two-way interaction between condition and test timing. Participants maintained their location accuracy after a night of sleep compared while their accuracy significantly declined after a comparable time awake. (*E*) Performance difference over time is plotted (the two-way interaction). In this graph, dotted bars represent training, solid bars represent test. In the *Top* row, within condition, lighter colors represent training 1 and darker represent training 2. In the second row, within condition, lighter colors represent test 1 while darker colors represent test 2. Asterisks denote main effect of training (Fig. 1*A*) and test (Fig. 1*E*) *p*’s <0.05.

We used a within-subject design to assess the relative contributions of sleep to spatial memory and navigation. We first evaluated baseline learning performance across the two conditions (Fig. 3 *A*–*C*). We did not find a difference between conditions in the time spent exploring to find the chests [*F*(1, 28) = 0.334, *P* = 0.568], but did find a main effect of training session [*F*(1, 28) = 19.856, *P* < 0.001, η^2^ = 0.415; Fig. 3*A*]. Participants became significantly faster in training 2 than in training 1 (*P* < 0.001). We also found no interaction between condition and training session [*F*(1, 28) = 2.510, *P* = 0.124, η^2^ = 0.082]. We found no main effect of condition for the number of chests opened while searching for each unique object [*F*(1, 28) = 1.696, *P* = 0.203, η^2^ = 0.057], training session [*F*(1, 28) = 0.047, *P* = 0.830, η^2^ = 0.002], or interaction between condition and training session [*F*(1, 28) = 0.818, *P* = 0.374, η^2^ = 0.028; Fig. 3*B*].

### Training Navigation.

For both conditions, we evaluated the total steps taken in each training session (results not plotted). We found no difference in total distance traveled across conditions [*F*(1, 28) = 0.145, *P* = 0.709, η^2^ = 0.005]. However, we did find that participants’ total distance traveled significantly differed across trainings [*F*(1, 28) = 23.278, *P* = <0.001, η^2^ = 0.454] as participants took fewer steps in training 2 (M = 886.65, SE = 47.259) than training 1 (M = 1186.142, SE = 47.259), indicating a faster learning rate with practice. We also did not find an interaction between condition or training [*F*(14, 28) = 2.935, *P* = 0.098, η^2^ = 0.095].

### Participant’s Average Test Time across Conditions.

**Fig. 4.**
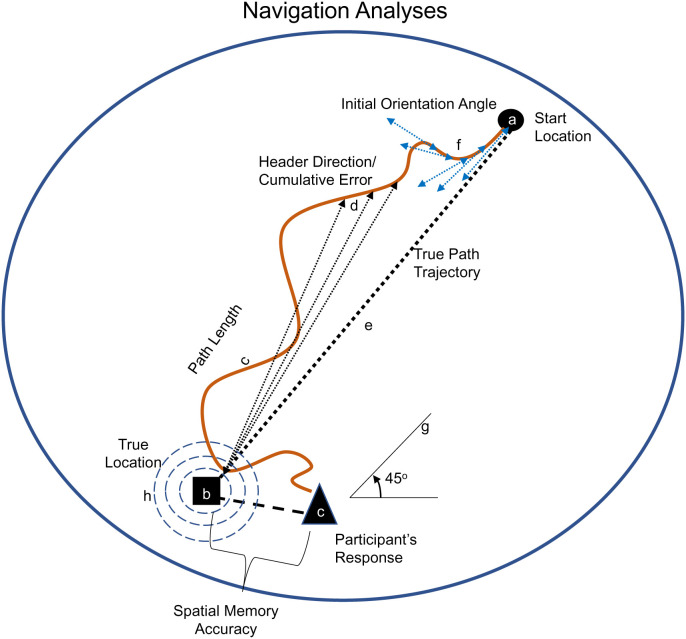
Navigation analyses. An example path is illustrated in orange with the navigation analyses explained. 1) Spatial memory accuracy was measured as the Euclidian distance between the true location (*B*) of the object and the participants’ placement of the object at test (*C*). 2) Path length was the total number of steps between the start location (*A*) and participant’s placement of the object (*C*). 3) Header direction was the average angle (three example angles presented at *D*) in which the Minecraft character was directed at each path step between the start location (*A*) and the true object location (*B*) until the participant placed the object (*C*). Cumulative header direction was the summation of the error (all *D* example arrows added at each path step) with lower numbers representing more direct paths. 4) Initial orientation angle (*F*). We averaged the angle oriented for the first five steps (angle of arrows in blue) for each object trial as a proxy for an allocentric sense of direction. 5) Approach angle (*G*). We calculated the angle with which participants approached the object at training and compared it to the approach angle at test. Participants had a 45° view of the world at all times, thus within this range was considered the same viewpoint. 6) Search time within proximity (*H*) was the total number of steps taken within concentric circles of radii at 10, 20, and 30 blocks near the true object location.

In line with prior sleep-dependent spatial research outcomes, we first evaluated the total time spent in the test phases. We found no main effect of condition [*F*(1, 28) = 0.166, *P* = 0.686, η^2^ = 0.006; test 1 plotted in Fig. 4 *C*, *Left*]. We did find a main effect of test [*F*(1, 28) = 14.697, *P* = 0.001, η^2^ = 0.344], with participants being significantly faster in chest placement at test 2 than test 1 (not plotted). We did not find a significant interaction between condition and test time [*F*(1, 28) = 0.280, *P* = 0.601, η^2^ = 0.01].

### Participant Spatial Location Accuracy.

We evaluated participants’ memory accuracy of chest locations at two timepoints: immediately after learning (test 1, Fig. 3 *C*, *Left* for test 1 only) and 12 h later after a period of sleep or wake (test 2) (see Fig. 3*D* for test 1 and 2 plotted together and Fig. 3*E* for differences over time plotted). We did not find a main effect of condition [*F*(1, 28) = 0.016, *P* = 0.902, η^2^ = 0.001] or test [*F*(1, 28) = 0.001, *P* = 0.98, η^2^ = 0.0] but did find a significant interaction of condition by test [*F*(1, 28) = 4.981, *P* = 0.035, η^2^ = 0.149; Fig. 3*D* for plotted tests]. Participants had a significant difference between conditions in spatial location accuracy between test 1 and test 2 (Fig. 3*E* for difference plotted), with participants’ performance maintained after a period of sleep (M = 1.83, SD = 7.09) but declining after a period of wake (M = −1.86, SD = 5.55). Within conditions, we did not find a significant difference in performance from test 1 to test 2 (*p*’s >0.081).

### Participant’s Global Spatial Navigation.

We computed a series of navigation analyses using participants’ average scores to assess possible changes between conditions and across tests (see Fig. 4 for navigation analyses conducted and *SI Appendix*, Fig. 1 for two individual participants’ navigation performance from start location [one of the four corners] to chest placement).

**Fig. 5.**
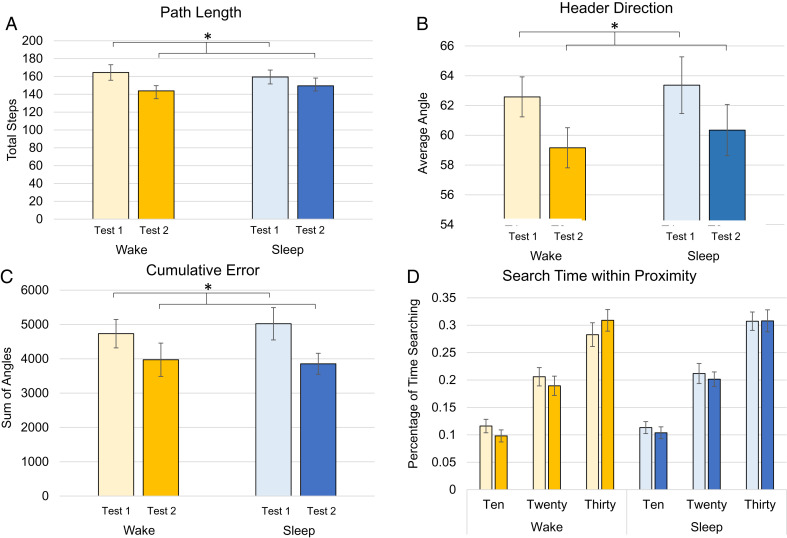
Global navigation analyses. Across all navigation analyses, we found a significant difference of test session, with improvement at test 2 compared to test 1, but no effect of condition. Plotted are (*A*) path length, which is the average number of steps between the start location and the participant’s object placement. (*B*) Header direction is the average angle at each step between the start location and the true object location. (*C*) The cumulative error is the summation of each angle between the start and true location. This takes into account when participants have turned around and are headed in the incorrect direction, with higher scores representing greater error. (*D*) Participant’s search time percentage within proximity of the true location is plotted for each radii of 10, 20, and 30 blocks away from the true location at each test. Within conditions, all light colors represent test 1 and darker colors represent test 2. Asterisks denote main effect of test *p*’s <0.05.

We first evaluated participants’ average distance traveled, termed path length, when placing the objects (Fig. 5*A*). We found a main effect of test [*F*(1, 28) = 5.761, *P* = 0.023, η^2^ = 0.171] with participants walking less during test 2 than test 1. We did not find a main effect of condition [*F*(1, 28) = 0.002, *P* = 0.963, η^2^ < 0] or interaction between condition and test [*F*(1, 28) = 0.537, *P* = 0.47, η^2^ = 0.019].

We followed this by analyzing participants’ average header direction accuracy as a global average (Fig. 5*B*) and cumulative error score (Fig. 5*C*), finding similar testing navigation outcomes to total time. For the average header direction, we found a main effect of test [*F*(1, 28) = 5.868, *P* = 0.022, η^2^ = 0.173], with participants’ header direction more accurately angled toward the true chest location at test 2 than test 1, but no main effect of condition [*F*(1, 28) = 0.471, *P* = 0.498, η^2^ = 0.017] or interaction between condition and test [*F*(1, 28) = 0.008, *P* = 0.928, η^2^ = 0; Fig. 5*B*]. For the cumulative error score, which better takes into account when participants are angled the wrong direction, we found a main effect of test [*F*(1, 28) = 5.930, *P* = 0.022, η^2^ = 0.175], but no effect of condition or interaction (*p*’s >0.531, η^2^’s <0.014, Fig. 5*C*), further evidence that participants maintained their accuracy on test 2 compared to 1. We then evaluated participants initial orientation angle, which was the average header direction of each of the first five steps. In essence, this is a proxy for an allocentric sense of direction, as it assesses participants’ ability to immediately orient themselves within the first few steps after teleporting and being instructed to find a specific object. We found no effect of condition [*F*(1, 28) = 2.717, *P* = 0.110, η^2^ = 0.088] nor an interaction between condition and test [*F*(1, 28) = 0.003, *P* = 0.958, η^2^ = 0.0]; however, we did find a significant effect of test [*F*(1, 28) = 4.338, *P* = 0.047, η^2^ = 0.134].

We also evaluated whether participants spent their time “searching” in the correct location by determining the percentage of time spent near the real chest location at close (within a 10-block radius) and far (within a 30-block radius) proximity (Fig. 5*D*). We ran a repeated measure analysis of variance (rmANOVA) of condition (sleep, wake), radii (10, 20, 30), and test (test 1, test 2). We found a main effect of radii [*F*(2, 56) = 610.677, *P* < 0.001, η^2^ = 0.956] but no main effects of condition or test (*p*’s >0.124) or interactions among the factors (*p*’s >0.374). Post hoc analyses revealed participants spent a significantly greater percentage of their time in the increasingly larger concentric circles around the true locations, (radii 10: M = 0.108, SE = 0.008; 20: M = 0.202, SE = 0.012, 30: M = 0.302, SE = 0.014; *p*’s <0.001).

### Participant’s Object-by-Object Spatial Navigation.

We evaluated spatial navigation at the object-by-object level. In line with participants’ average navigation measures, we found similar effects for path length, with a main effect of test [*F*(1, 340) = 6.695, *P* = 0.010, η^2^ = 0.019], but no effect of condition [*F*(1, 341) = 0.901, *P* = 0.343, η^2^ = 0.003] or interaction of condition by test [*F*(1, 341) = 1.026, *P* = 0.312, η^2^ = 0.003]. We found a similar pattern for header direction, with a main effect of condition [*F*(1, 340) = 10.495, *P* < 0.001, η2 = 0.030] and test [*F*(1, 340) = 11.683, *P* < 0.001, η^2^ = 0.033]. Participants in the wake condition were more direct (*P* = 0.001, M_S_ = 63.949, SE = 0.791, M_W_ = 60.816, SE = 0.668). We did not find an interaction of condition and test [*F*(1, 340) = 0.303, *P* = 0.583, η^2^ = 0.001].

### Relationships between Global Spatial Memory and Global Navigation.

We first evaluated whether there were relations between participants’ average spatial memory and navigation measures by condition (*SI Appendix*, Table S1). For the sleep condition, we found that spatial location accuracy and path length did not correlate at either test 1 (*r* = −0.21, *P* = 0.258) or test 2 (*r* = −0.34, *P* = 0.069). The spatial location accuracy also did not correlate with the header direction at test 1 (*r* = 0.35, *P* = 0.063); however, it did correlate with test 2 (*r* = 0.38, *P* = 0.04). Spatial location accuracy did not correlate with the initial orientation angle at test 1 or test 2 (test 1: *r* = −0.147, *P* = 0.446, test 2: *r* = 0.166, *P* = 0.389). Header direction and initial orientation angle did not correlate at test 1 (*r* = 0.055, *P* = 0.775) but did correlate at test 2 (*r* = 0.432, *P* = 0.019). Path length and header direction did not correlate at test 1 (*r* = 0.34 *P* = 0.071) or test 2 (*r* = 0.14, *P* = 0.454). Path length and initial header direction did not correlate at either test.

The wake condition results paralleled those of the sleep condition (*SI Appendix*, Table S1). Participants’ spatial location accuracy and path length did not correlate at test 1 (*r* = −0.135, *P* = 0.484) or test 2 (*r* = 0.069, *P* = 0.722). Spatial location accuracy also did not correlate at test 1 with header direction (*r* = 0.343, *P* = 0.068) but did at test 2 (*r* = 0.436, *P* = 0.018). Path length and header direction correlated at both test 1 (*r* = 0.511, *P* = 0.005) and test 2 (*r* = 0.491, *P* = 0.007). Path length and initial header direction did not correlate at either test.

### Relations between Spatial Memory and Navigation at Finite, Object-by-Object Level.

**Fig. 6.**
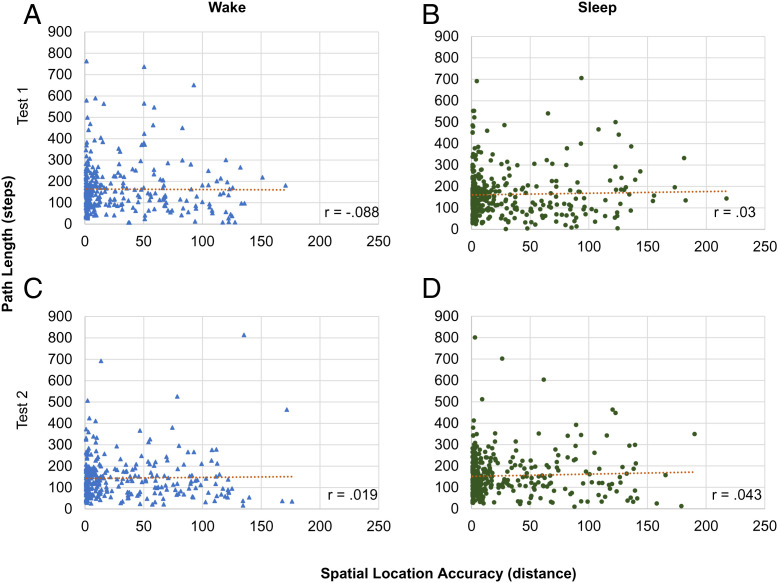
Path length and spatial memory analysis at the object-by-object level. In neither condition did object memory and path length correlate (*A–D*). Here we show the nonsignificant relationships for both conditions between the difference in memory performance from test 1 to test 2 and the difference in path length from test 1 to test 2.

**Fig. 7.**
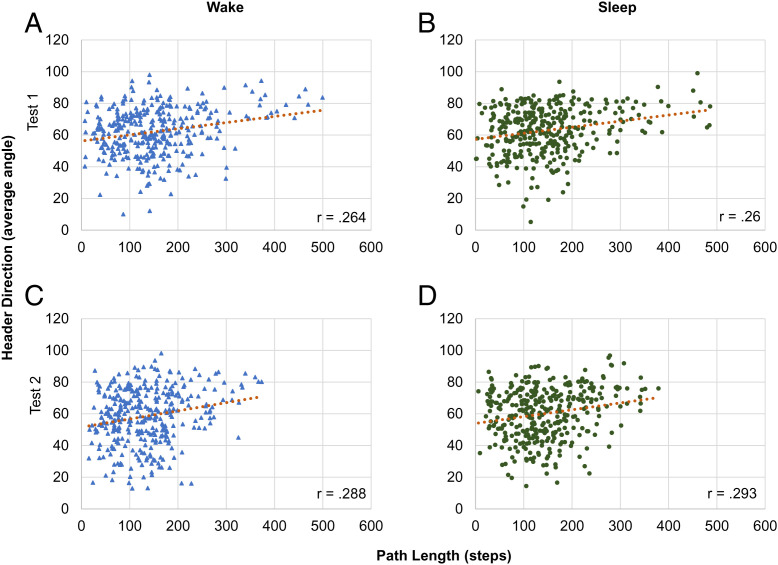
Header direction and spatial memory analysis at the object-by-object level. Header direction is the angle moving at the start of test trial with 0 indicating the best fit line from starting position to true object location. Header direction correlated positively with path length for both conditions at each test, demonstrating that participants with better orientation for the true object location took fewer steps prior to placement.

We then collapsed all participants’ data and investigated navigation measures at an object level by condition (see *SI Appendix*, Table S2 for all correlations and Figs. 6 and 7 for plots). In the sleep condition, for all objects, the spatial location accuracy did not correlate with path length at either test 1 (*r* = 0.03, *P* = 0.582; Fig. 6*B*) or test 2 (*r* = 0.043, *P* = 0.423; Fig. 6*D*). Spatial location accuracy did not correlate with the initial orientation at test 1 or test 2 (test 1: *r* = 0.088, *P* = 0.103; test 2: *r* = 0.097, *P* = 0.073). However, spatial location accuracy did correlate with header direction at both tests (test 1: *r* = 0.220, *P* < 0.001; test 2: *r* = 0.198, *P* < 0.001), with worse memory performance correlating with worse angle direction. Path length and header direction positively correlated at test 1 (*r* = 0.260, *P* < 0.001) and test 2 (*r* = 0.293, *P* < 0.001, Fig. 7*B*). Header direction and initial orientation angle correlated at both test 1 and test 2 (test 1: *r* = 0.576, *P* < 0.001; test 2: *r* = 0.633, *P* < 0.001). Path length and initial orientation correlated at both test 1 and test 2 (test 1: *r* = 0.123, *P* = 0.022; test 2: *r* = 0.232, *P* < 0.001).

These associations were nearly identical in the wake condition. Participants’ spatial location accuracy did not correlate with path length at either test 1 (*r* = −0.008, *P* = 0.884) or test 2 (*r* = 0.019, *P* = 0.725; *SI Appendix*, Table S2 and Fig. 6 *A* and *C*). Header direction did not correlate with the spatial location accuracy at test 1 (*r* = 0.07, *P* = 0.196) but did correlate at test 2 (*r* = 0.230, *P* < 0.001). Spatial location accuracy did not correlate with initial orientation at test 1 (*r* = 0.028, *P* = 0.611) but did correlate at test 2 (*r* = 0.168, *P* = 0.002). Header direction and path length positively correlated at both test 1 (*r* = 0.264 *P <* 0.001) and test 2 (*r* = 0.288, *P* < 0.001; Figs. 7*A*). Header direction and initial orientation correlated at test 1 and test 2 (test 1: *r* = 0.565, *P* < 0.001*;* test 2: *r* = 0.599, *P* < 0.001). Path length and initial orientation did not correlate at test 1 (*r* = 0.104, *P* = 0.055), but did correlate at test 2 (*r* = 0.148, *P* = 0.006).

### Exploratory Analysis of Approach Angles at Object-by-Object Level.

We then ran exploratory analyses to investigate whether participants’ approach angle (angle and vantage point that participants’ characters approached chests during trainings and tests) influenced long-term memory. All correlations were Bonferroni corrected within condition (*P* = 0.008) and we report what withstood correction. If the approach angle between trainings and subsequently tests correlated, it would suggest that participants approached objects from similar vantage points, which might be a possible underlying memory strategy. For the sleep condition, we found that the approach angle between trainings correlated (*r* = 0.238, *P* < 0.001). The approach angle correlated between training 2 and test 2 (*r* = 0.180, *P* < 0.001). Test 1 and test 2 approach angles also correlated (*r* = 0.145, *P* = 0.007). Altogether, this suggests that participants approached the objects from similar vantage points, especially between training 2 and test 2, which is a potential memory strategy.

During wake, we found that the approach angle correlated between trainings (*r* = 0.309, *P* < 0.001). Test 1 approach angle correlated with the training 2 approach angle (*r* = 0.223, *P* < 0.001). Test 2 approach angle correlated with training session 1 (*r* = 0.183, *P* < 0.001). The approach angle correlations between test 1 and test 2 did not withstand correction (*r* = 0.111, *P* = 0.04). Altogether, this suggests that the participants were not consistent in approaching the objects from similar vantage points between training and test sessions.

## Discussion

Our goals were to investigate how spatial memory accuracy and navigation changes over time within an individual and to disentangle the contributions of sleep-dependent mechanisms to spatial memory and navigation. As the term spatial memory is widely used, in our paper, we specifically define the term to be the retention of metrically accurate locations of objects. To this end, we designed an ecologically valid, remote spatial task that combines free exploration in an open arena, spatial memory recall, and navigation assessment using a popular video game, Minecraft. Our results showed that participants’ spatial memory for specific locations benefitted from a night of sleep by maintaining its accuracy, but declined across an equivalent period of wake. We, however, did not find a sleep-specific benefit for navigation when analyzed at both global (average) and finite (object-by-object) levels. Our results suggest that sleep-dependent mechanisms primarily support the retention of an underlying, allocentric cognitive map but do not influence navigation-specific behaviors.

### Spatial Memory and Navigation.

Extending prior sleep research, we measured participants’ precise object-location memories, finding a sleep-dependent improvement in accuracy. Earlier studies had similarly shown sleep-dependent benefits in navigation time and route accuracy to designated endpoints (31, 32, 33, 35). However, time is unlikely to be the most sensitive measure for spatial memory as pauses during navigating to recall or evaluate landmarks or game-based movement speed could impact the data (32). Similarly, route accuracy tests spatial memory for single predesignated endpoints, thus is unable to ascertain the metric specificity of an individual’s underlying hippocampally dependent cognitive map. In evaluating the contributions of sleep to hippocampally dependent spatial memory aspects, Noack et al. (33) showed that after sleep, explicit awareness of their intentionally regionalized environmental structure improved. Following this study, Noack et al. (34) further showed that participants’ ability to flexibly use landmark or boundary cues to place objects improved with sleep. However, they did not find a sleep-dependent improvement in the metric accuracy of object placement in line with our findings. This null finding is likely the result of the task and environment structure. Objects were learned in isolation in either the presence of a landmark or environment boundary, which was then switched at test. In comparison, we attempted to create an ecologically valid environment in which the participant had the potential to form a strong cognitive map through free exploration of the open arena. In our task, participants learned the spatial relationships between all the trained objects, landmarks, and the environment boundary. Thus, our study highlights the contribution of sleep-dependent mechanisms to support the hippocampal-dependent retention of allocentric environmental representations.

We also demonstrated that navigation does not appear to be supported by sleep-dependent memory mechanisms. At both the global and finite levels, after both wake and sleep, we found navigational improvement from test 1 to test 2. This improvement was consistent across all navigation analyses, including total path length, header direction, and initial orientation angle. Again, one potential reason for the discrepancy between our null results and other prior sleep-dependent research is how our tasks diverged in assessing spatial cognition. For our MMN task, we did not have a designated endpoint, such as the exit of a maze or a landmark, and instead, our participants’ “endpoints” were their subjective object placements based on their internalized environment representations. How they navigated to these positions could be done through allocentric or egocentric navigation strategies, or in some combination of the two (21). As previously discussed, Noack et al. (34) showed that participants were able to flexibly navigate to place an object in an open arena despite a change in the presence of a landmark or event boundary between the learning and test phases of their task. As we did not alter landmarks or boundaries, or query participants’ navigation strategies, it is not clear whether participants chose to use allocentric or egocentric navigation strategies in our task. Our hint as to a strategy participants might employ comes from our exploratory analysis of participants’ approach angles between learning and test object placement. Participants in the sleep group appeared to be more consistent in having similar vantage points during the placement of objects than they did in the wake group. This could potentially point to participants attempting to engage a strategy of pattern matching between the memory of learning the original location at a specific vantage point and subsequently during testing. Whether this approach angle signals the navigational choice to engage allocentric or egocentric strategies is less clear.

Although we did not find a relation between object-location accuracy and path length, we did find an association between object-location accuracy and header direction. Header direction and path length were also strongly correlated. In our task, header direction was calculated as the angle variation at every step between the starting point and the actual object location, rather than participants’ subjective object-location placement. Thus the correlation patterns between the object-location accuracy (the “true” spatial memory) and header direction (the connected spatial memory and navigation metric) is unsurprising, as is the correlation between the header direction with path length (the true spatial navigation measure). The more independent spatial measures, object-location accuracy and path length, did not correlate. Over the delay, in both conditions, the header direction and object-location accuracy remain correlated. In addition, in the wake condition, the initial angle oriented also correlated with spatial location accuracy. Furthermore, at the item-by-item level, participants’ header direction was more direct than in sleep, possibly suggesting better retention of the environment to get to the vicinity of the object being tested. Whether this suggests differences in navigation strategies is unclear, given that overall spatial location accuracy declined. This overall pattern between the two conditions is consistent with the hypothesis that sleep supports the consolidation of the allocentric spatial representations, but does not support navigation.

In line with current theories articulating the support of sleep for hippocampally dependent information (1), it is not surprising that we did not find a sleep-specific benefit for navigation. Navigation is a dynamic behavior that integrates a wide range of cognitive processes that are not known to be sleep dependent. Although we found no specific role of sleep, it is not surprising that other prior sleep-dependent studies using navigation-based metrics for spatial memory did (31–33, 35). Distinguishing navigation from memory is complicated, as it is a core component of this dynamic process; yet, our task design supported our ability to disentangle the contributions of sleep to navigation more so than past tasks.

### MMN Task Development.

While video games have commonly been used in scientific research to train, intervene, and assess various cognitive abilities and brain processes (42–45), here we used the video game Minecraft as a platform for remote, behavioral research. Previously, the video game Minecraft was used as a proxy for environmental enrichment in humans and used to observe individual exploratory behaviors (46–48). Our ability to create a task to disentangle the contributions of sleep to spatial memory and navigation are further testament to the game’s potential in studying naturalistic, ecologically valid cognitive processes.

There are clear benefits to using the video game Minecraft as a platform for behavioral research. First, Minecraft is a sand box video game, which means the world is open for a free-roaming experience with no winning or losing, no hard fast rules, and no right or wrong way to play the game. This provides an easy translation to research as participants are not required to follow preordained rules; rather, the within-Minecraft task structure can be designed by the researcher. Second, while Minecraft is not entirely open source, it can be customized and modified to track users movements, activities, and actions, as well as create tasks within the world of Minecraft. Lastly, Minecraft can be played remotely on custom servers (47, 48) that can track a significant amount of information about users’ behaviors and contexts. Remote and accessible testing platforms could be helpful for exploring sleep-dependent cognition; however, they are currently underutilized.

Additionally, one of the strengths of our task lies in its potential for comparison between human and animal spatial cognition. Our task parallels many of the features in animal open field exploration tasks in which animals explore an arena that can include distal and/or proximal cues while learning the locations of objects. In these tasks, navigation is evaluated and performance is measured by exploration when objects are moved or remain in consistent locations. In a paralleling study of sleep-dependent spatial memory study in rats, Binder et al. found that rats retained the location of objects better after a period of sleep, in line with prior human studies (49). Although they explored one-trial learning and two objects, Minecraft can similarly be scaled up or down, with distal and proximal cues reduced or enhanced. The Minecraft platform creates an optimal opportunity to lead translational studies comparing species’ cognition.

At present, there is a clear scientific initiative to increase accessibility and participation to populations not typically able to engage in in-person research studies. Moreover, with the ongoing and future COVID-19 challenges, maintaining participant safety while investigating key mechanistic questions is crucial. Although challenges exist in remote data collection, merging with wearable technology can meet the need for safe, physiologic assessments. Our MMN task successfully extended prior research to disentangle sleep’s contribution to spatial memory and navigation. Looking to the future, we believe that remote tasks, like ours, will be instrumental in repeated testing required to further disentangle individual longitudinal cognitive trajectories, such as those across development, aging, or disease.

### Limitations.

Lack of polysomnography limited our ability to assess sleep-specific mechanisms supporting spatial memory. Instead, we were required to rely on participants’ sleep diaries as measures of total sleep time. This coarse subjective measure did not correlate with any memory or navigation features. As such, future work should evaluate the relationship with sleep-specific features known to be critical in supporting hippocampal-dependent memory consolidation (1). We also designed our training criterion of finding all 12 objects or meeting a 10-min limit to maximize the likelihood that participants could find and learn all objects within an environment. Across both studies, only one subject met this limit in both trainings and two others met the 10-min limit at a single training. Depending on the goal of the task, future alterations could experimentally manipulate this training criterion to evaluate whether more or less time in the environments impacts spatial memory or navigation. We used a common methodological sleep/wake design to investigate change in performance over consolidation periods of wake or sleep. Although overall baseline performance was equivalent, we cannot rule out potential circadian effects in our study. However, in study 2, participants had equivalent rates of training in both conditions, i.e., time spent in trainings and the total number of chests opened in each training. This lack of difference between sleep and wake conditions in these training metrics makes circadian timing less likely to impact spatial environment learning. As we did not evaluate sleep until the night of the study, we also cannot speak to how sleep patterns leading up to the study might have influenced overnight performance. Future studies can address this question. Our participants were also 75% female. Prior research has found sex differences in spatial navigation (28); however, it remains unclear whether sex differences would be present in sleep-dependent memory spatial memory. Additionally, we did not query participants’ use of allocentric or egocentric navigation strategies. Moving forward, understanding the strength of the underlying allocentric map and how it relates to changes in sleep-dependent spatial memory performance and navigational behaviors may provide insight into the development of cognitive maps and navigation strategies. We also did not assess subjects’ conscious navigation strategies, thus are unable to identify whether they used allocentric or egocentric strategies. Further, after wake, participants’ header direction improved more so over the delay than over sleep. It is unclear why we found this, given that spatial location accuracy declined. Future studies should further investigate this change in performance and whether it suggests differences in navigation strategies that develop across wake compared to sleep. Another limitation of our study was internet stability. We lost nine participants’ data due to internet connection issues as it altered the speed at which participants could explore the world, minimized the ability to see the entire 45° screen of landmarks, and caused disjointed movement. While generally remote studies increase access, altering task demands due to slow or frequently disconnecting internet can alter memory encoding. Additionally, while we attempted to control participants’ environments by requesting they be alone, without distraction, and focused on the task, home-based distractors are present and uncontrollable. Similar to our remote study protocol, future researchers should continue to set required experiment boundaries of participants’ environments. Lastly, it is important to note that we, and others, use virtual tasks as a proxy for real navigation. We cannot rule out that there may be differences between navigating in the real world compared to virtually.

## Conclusions

How sleep impacts spatial memory and navigation remain difficult to study in humans. We developed a remote sleep-dependent Minecraft Memory and Navigation task to assess the retention of underlying, allocentric cognitive-map relations and navigation-specific behaviors. We found that the metric accuracy of object locations benefitted from a night of sleep compared to wake. We did not, however, find a benefit of sleep for spatial navigation. Our findings are consistent with the hypothesis that sleep benefits hippocampal-dependent aspects of spatial memory, namely, the retention of the spatial relationships within an environment. Virtual remote tasks like the one we developed create a path for investigating these intertwined processes and support the initiative to increase accessibility and participation to populations not typically able to participate in in-laboratory studies. Looking to the future, we believe that the creation of a sleep-dependent spatial task with repeated and remote testing potential will likely be instrumental in disentangling individual longitudinal trajectories, such as those across development, aging, or disease.

## Materials and Methods

### Sample.

A total of 130 healthy participants (99 female, mean age: 22.24, SD = 3.72 y) participated in studies 1 and 2. Prior to participation, participants were administered verbal and written informed consent. Study procedures were approved by the Institutional Review Board at the University of California, Irvine. Participants were recruited from the community via online flyers and from the student research pool. In study 1, participants received course credits and in study 2, participants received monetary compensation for their time. Participants were required to have consistent sleep/wake cycles, going to sleep prior to 2 AM and waking before 10 AM, and be generally healthy, with no preexisting psychological or medical conditions. Self-rated novice or intermediate Minecraft players were recruited from the community and university-based research participation pools.

### Study 1 Methods.

#### Participants.

Eighty-six participants were administered our Minecraft task to evaluate the Minecraft environments and equate baseline performance. Two participants did not complete the test portion of the task and were excluded from the data analysis.

#### General Minecraft Memory and Navigation task procedures.

At encoding, participants learned the locations of 12 unique objects within chests hidden around the environment (see Fig. 1*A* for MMN environments, Fig. 1*B* for example chest hidden in the ground, and Fig. 1*D* for MMN task timeline). Participants encoded the object locations during two identical encoding sessions—training 1 and training 2—through free exploration. Participants were explicitly told to find all 12 chests and to remember each object-chest location. A training session culminated when participants either found all 12 unique objects or had explored the environment searching for the objects for 10 min. As such, there was the potential that participants could visit some object chests more than once within a training session. After one of the training conditions was met—finding all objects or searching for 10 min—participants would enter the next phase of the task via a start room. During training 1 and training 2, all chests were marked with a semitransparent pink block floating one block above a chest to mark its location (see Fig. 1*B* for marked chest).

For study 1, only test 1 was conducted; however, both test 1 and test 2 were identical in methodology except for the delay in timing; test 1 was conducted immediately following training 2 and test 2 was conducted 12 h later after a period of wake or sleep. At test, participants randomly spawned in one of four locations located at outer corners of the environments. Across all environments, these test spawn locations were equivalent and equidistant from the nearest objects. Once spawned, participants were prompted to place a chest for a specific object in the location they believed it was located during training sessions (see Fig. 1*C* for tested object prompt). All 12 objects were tested in each test, with the order randomized per participant. To note which object was being tested, participants read written instructions on the bottom center of the screen and saw the item in their inventory at the bottom of the page. Participants were allotted 180 s per chest/object placement. Once all objects were placed, a test was completed. Please visit https://www.youtube.com/watch?v=sgLC8_hDin8 if interested in watching a short video depicting our practice world for both training and testing segments.

### Environment Designs.

We designed four novel, open-arena Minecraft environments of equal size (see Fig. 1*A* for environment images). Within each environment, 12 chests were located in an equidistant grid within the ground. Each of the four environments had unique landmarks, color schemes, and block types. Environment 1 was modeled after a desert environment, environment 2 was modeled to be lush green and resembled rolling fields, environment 3 was modeled to be a winter environment with snow, and environment 4 was modeled to be drought-like, with dry grass and gray mountains (see Fig. 1*A* for a bird’s eye view of the four environments). In addition, landmarks were constructed within each environment to mimic the natural Minecraft landscapes but modified enough to be distinct and provide participants with subtle positional references. These landmarks include features such as a large mountain made from a specific block type, to areas of tall trees, flower and plant life, streams and bodies of water, and more. Each environment’s 12 chests contained a unique object (examples including axe, clay pot, feather, gold bar, bottle, etc.) Objects were not repeated across environments. In order to prevent participants from wandering beyond the testing area surrounding the chests, a boundary wall around the arena was constructed to limit player escape. This boundary wall was consistent across environments in architecture and transparency, allowing participants to observe areas and landmarks outside the wall. Normal Minecraft spawns (i.e., animals) and time synchronized features were disabled for the duration of this study to ensure equal experience across participants.

### Practice Environment.

Prior to the first time engaging in the task, all participants were informed of the game mechanics and task instructions. A practice environment was designed to acclimate and ensure an equitable understanding and capacity for the task. The practice environment resembled the four test environments in appearance; however, in order to limit pretask exposure time, the practice environment contained four chests with unique objects (anvil, sunflower, sea pickle, and fire coral chests). Participants were instructed to practice moving through the Minecraft world and were administered a shortened version of the task, including a single training trial and single test. Researchers provided feedback to participants such that all participants had equivalent performance in practice. Once participants had progressed through the practice environment, they were administered the task.

### Minecraft Servers.

All Minecraft participants played on individual custom servers, hosted on a local server in the laboratory. Custom servers were created using SpigotMC (https://www.spigotmc.org) and each server contained two custom mods. Tracker (created by G.D.C.) is a mod that tracks spatial locations of individuals and information about the world they created (blocks placed, current biome, time spent underground, etc.). The Minecraft Memory and Navigation task (created by K.C.S. and G.D.C.) is a mod that contains the task used for this study. Each mod creates an output log file that is updated in real time immediately upon log in of the server. All participants were explicitly instructed while in the game to not intentionally kill their Minecraft characters. Although Minecraft does have “lives” associated with the game, as can be seen denoted by hearts on Fig. 1*C*, we did not use this feature during the task and all participants used the same originally spawned character for the duration of the task. Lastly, we did not control for participants choice of walking or running during the trial as this should only influence trial and test time, but not spatial memory accuracy or navigation metrics of path length, header direction, or cumulative error.

### In-Person and Remote Study Procedures.

With the onset of the COVID-19 pandemic, scientific laboratories were closed. As such, data were collected entirely remotely for study 1 and remotely for study 2, except for six initial participants. Procedures were exactly the same for in-person and remote assessments except that remote participants completed assessments in their home via Zoom and temporarily provided Minecraft accounts. No differences were found in study 2 when comparing in-person and remote data collection, thus data were combined. For each remote participant, we provided instructions to download Minecraft onto participants’ personal computers, provided temporary accounts, and supported participants via Zoom with the downloads and setup. Participants also watched a short instructional video on the MMN task prior to participation. During the testing sessions, participants met with research assistants via Zoom where they were provided general MMN task instructions and practiced the MMN task (see below for details). Participants shared their screens via Zoom screen share for the duration of participation so research assistants could monitor participants’ task activity. Participants were not allowed to play Minecraft in between testing sessions and provided accounts were temporarily disabled to prevent any use in between.

### Statistical Analysis.

#### Environment assessment.

Our main variables of interest in determining the environments’ equality were participants’ learning rate, specifically the number of objects found (i.e., number of chests opened) during each training, the time spent searching in each training, and the spatial location accuracy, which was the distance between the participant’s object placement during the test and the original, learned location using Euclidean measurement. We used a rmANOVA to evaluate within-subject factors of training (training 1 and training 2) and between-subject factor of environment (Env1, Env2, Env3, and Env4). We used one-way ANOVAs to evaluate chest location accuracy differences. Secondary variables of interest were the time spent within the training and test task phases. Timing was chosen as a secondary exploratory variable in evaluating the environments as participants had the freedom to both walk or run through the environments. Given this, participants’ pace was likely less consistent within and across phases (e.g., participants may have walked quickly at times but not others or some participants may have walked quickly the entire time while others slowly). Analyses were conducted in SPSS. Alpha of 0.05 was considered for significance.

### Study 2 Methods.

#### Study design.

We used a within-subject design to assess the relative contributions of sleep to spatial memory and navigation (see Fig. 1*E* for timeline). Study 2 was conducted entirely remotely except for six subjects (see study 1 for remote study procedures). No differences were found in study 2 when comparing in-person and remote data collection, thus data were combined. Participants were counterbalanced across conditions and environments. For the sleep condition, participants were trained and tested (test 1) ∼2 h prior to bedtime, typically between 8 and 10 PM and were tested again (test 2) 12 h later, ∼1 h after waking up. In contrast, when undergoing the wake condition, participants were trained and tested (test 1) ∼1 h after waking up (typically between the hours of 8 and 10 AM) and were tested again (test 2) 12 h later. Participants also completed a sleep diary and were instructed not to nap during the wake condition.

#### Participants.

Forty-four participants were remotely administered our Minecraft task with a second test administered after a 12-h delay filled with either sleep or wake. We conducted an a priori power analysis in G*Power using repeated measures ANOVA sensitivity analyses, which identified that an *n* = 24 has 80% power to detect a small within-person effect on memory (Cohen’s f = 0.25). Given the nature of remote research, we recruited more subjects than required. We analyzed 29 subjects in our final sample as 15 participants were excluded due to internet issues, not completing both conditions, inability to follow task instructions, and 3 were outliers 2.5 SDs above the mean in learning and memory performance. Participants self-rated as novice (*n* = 21) or intermediate (*n* = 7) players, with one self-rating as an expert. Lastly, 6 participants in the final sample did not complete the sleep diary or questionnaires.

#### Questionnaires.

Participants were administered a remote sleep diary and questionnaires via Qualtrics. The sleep diary consisted of bed and wake times, number and timing of arousals, and sleep quality. With these variables, we computed total sleep time (TST) and sleep efficiency (SE). Participants completed the sleep diary the night of the wake condition to confirm napping had not occurred between sessions. For the sleep condition, subjects completed a part of the sleep diary at night to gather bedtime and in the morning, to gather night sleep information and wake time. Research assistants confirmed sleep diary completion and checked time stamps. Participants were also administered a standard battery of questionnaires the week between the conditions that were not relevant to the direct study, including the Sleep Hygiene Index, the Center for Epidemiological Studies Depression Scale (CES-D), and the Horne-Ostberg Morningness-Eveningness Questionnaire (50–53). These questionnaire analyses are included in *SI Appendix*.

### Statistical Analysis.

#### Learning and memory analyses.

We used a within-subject design to evaluate whether sleep, compared to wake, supported spatial learning and memory. For baseline learning trajectories, we evaluated the time spent in each training phase and number of objects found (i.e., chests opened) until all 12 objects were found. We used a repeated measure analysis of variance (rmANOVA) of condition (sleep and wake) by training (training 1 and training 2) to evaluate each dependent variable. For spatial memory accuracy, our dependent variable was the Euclidian distance between the actual location of the object and the participants’ placement of the object at each test. We used rmANOVA of condition (sleep × wake) and test (test 1 and test 2) to evaluate participants’ average differences in object-location accuracy across the different consolidation periods. We also ran a separate rmANOVA to evaluate the average time spent in the tests phases with condition (sleep × wake) and test (test 1 and test 2).

#### Navigation analyses.

We evaluated participants’ spatial navigation in two ways, at averaging across items and at an item-by-item level (see Fig. 4 for analyses graphically presented). This provided us the opportunity to assess participants’ overall navigation patterns and to then explore differences in navigation patterns dependent on the spatial location and memory strength of each item’s object memory (i.e., weakly or strongly remembered objects). At the item-by-item level, we removed objects 2.5 SDs away from the mean for memory performance and objects not tested in one test or the other due to Minecraft glitch for either condition. Our primary navigation analyses were path length, header direction, cumulative error, and initial orientation angle. Our exploratory analyses were the approach angle and search time within proximity of the true location. In training, path length is defined as the total number of steps walked within an entire training trial (Fig. 4*C*). At test, path length was the total number of steps taken between test trial start and chest placement. Header direction was a measure of how direct an individual’s path was between the start location and when they placed the test trial object (Fig. 4*D*). We computed header direction by averaging each angle the Minecraft character was oriented at every step between the start location and true location until the participant placed the chest. A header direction angle of 0° signifies a straight line to the true location while a positive or negative trajectory number indicates movement off the true path trajectory. We statistically analyzed the header direction using the absolute value off the true path trajectory. Cumulative error was calculated as the summation of header direction angle at every step between the start and true response, until participant object placement (Fig. 4*D*). This was to account for any backtracking (for example movement north then immediate movement south) as in header direction, this change in direction would be mathematically cancelled out, while in cumulative error, this would result in a higher cumulative score, i.e., more error. Thus, lower cumulative scores are better and suggest a more direct path during navigation. Initial orientation angle was the average header direction of the first five steps. In essence, this is a proxy for an allocentric sense of direction, as it assesses participants’ ability to immediately orient themselves from a teleported location demonstrating their ability to orient themselves for successful navigation (Fig. 4*F*). For exploratory analyses, we measured the approach angle, which we calculated as angle in which participants approached an object at trainings and compared it to the approach angle at test. In general, participants had a 45° view of the world at all times, thus an angle 22.5° more or less was considered within the same viewpoint (Fig. 4*G*). We also measured search time within proximity to the true locations of objects, similar to probe trials commonly used in the watermaze (44) (Fig. 4*H*). We evaluated the percentage of time spent within concentric circles surrounding the true object locations at test in increasing radii of 10, 20, and 30 blocks.

To evaluate the relationship between spatial memory accuracy and navigation measures, we ran Pearson *r* correlations. Analyses were conducted in SPSS. Alpha of 0.05 was considered for significance except for correlations, in which Bonferroni correction was applied and the corrected alpha is identified.

## Supplementary Material

Supplementary File

## Data Availability

Data are available at https://osf.io/b6tfz/. MNN task code: General code is available at https://osf.io/b6tfz/. Cognitive data have been deposited in Open Science Framework (https://osf.io/b6tfz/) (54).
