# Supplementary material for: Sleep facilitates spatial memory but not navigation using the Minecraft Memory and Navigation task
Source: Proc Natl Acad Sci U S A. 2022 Oct 17;119(43):e2202394119. doi: 10.1073/pnas.2202394119 (PMC9618094; doi:10.1073/pnas.2202394119)
Supplement: Supplementary File [file pnas.2202394119.sapp.pdf]

Supplemental Materials

Video Game Expertise

To assess for possible differences between novice (n = 21) and intermediate (n = 7) groups, we ran unpaired t-tests on spatial location accuracy performance difference within conditions. We found no significant differences in performance between groups for either condition (wake condition: t(26) = .9693, *p* = .3413; sleep condition: t(26) = .0539, *p* = .9574).

Spatial Navigation

Below is a figure panel of two examples of different participants' navigations at test. Participants randomly teleported to one of four locations during test and were instructed to place a specific object where it had been learned. As can be seen in Supplemental Figure 1, participants navigation pathways could be more or less direct.

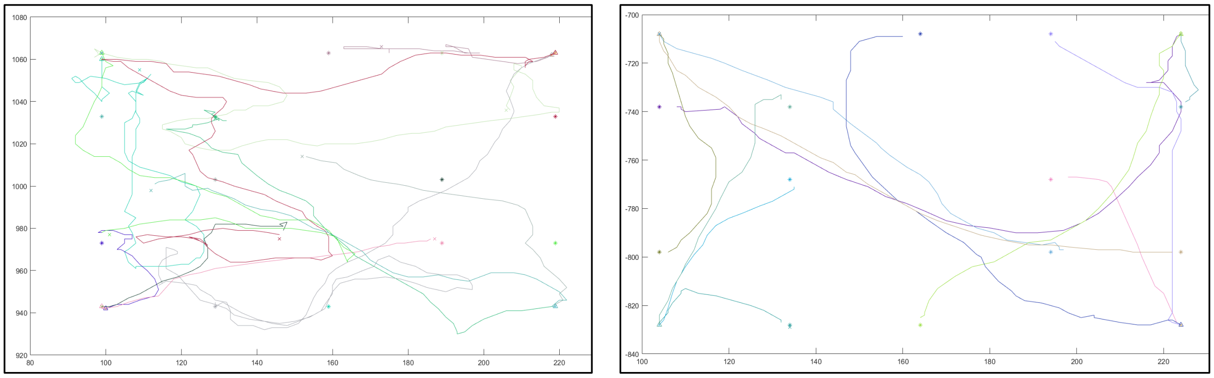

Examples of two different subjects' navigation at test

Supplemental Figure 1. Two participants' navigation through environments at test. At test, each object was administered by cued recall with written and visually-aided instructions (see Fig 1.C). At test, for each object, participants were teleported randomly to one of four corners of the environment and required to navigate using their memory to where they believed the object was located in training. Target locations are indicated by symbols. As can be seen on the left, some participants navigated extensively throughout the environment before placing the cued object. In contrast, others navigated more efficiently to where they placed the cued object.

Sleep Diary and Memory

Participants were administered sleep diaries during both conditions to confirm that participants slept during the sleep condition and did not nap during the wake condition. Sleep diary variables are reported in supplemental Table 1. We also analyzed within the sleep condition, if reported overnight sleep variables, including total sleep time, sleep onset latency,

and arousals, correlated with spatial memory performance measures. In the sleep condition, participants' average reported sleep time was 7 hours 18 minutes (SD = 65.7 minutes). We found no relationship between the participants' spatial accuracy at test 1 or test 2, nor the improvement overnight and reported total sleep time (p's > .136), arousal number (p's > .12), subjective feeling of being refreshed after sleep (p's > .479), or subjective sleep quality (p's >.613).

Questionnaires

Participants completed a sleep diary on the night of the sleep condition to assess bedtime, waketime, total sleep time, arousals, quality, and feelings of being refreshed. On average participants went to bed at 11:05 PM (SD = 1:11) with a median time of 11:20 PM. On average participants woke up at 7:43 AM (SD = 0:59), with a median wake time of 8:00 AM. Participants had an averaged of 7 hours and 18 minutes total sleep time (SD = 1 hour 5 minute). Participants reported .95% sleep efficiency (SD = .03), had an average of .3 arousals (SD = .55). Participants reported feeling refreshed on average at a 4.69 (SD = 1.49) level on a scale of 0 to 5 with 5 being the most refreshed. Participants also rated their sleep quality an average of 4.91 (SD = 1.2) on a 0 to 5 scale, with 5 being the highest quality. On the wake portion of the sleep diary, all participants confirmed that they did not nap during the day between sessions.

We administered a standard battery of questionnaires to all Study 2 participants between conditions. On the Horne-Ostberg Morningness-Eveningness Scale, four participants scored as a morning type, three scored as evening types, and the remaining 16 scored as intermediate types. On the Sleep Hygiene Index, participants scored an average of 18.56 (SD = 4.04). On this scale scores range 0 to 52 with lower scores representing better sleep hygiene and higher scores representing worse sleep hygiene. On the Center for Epidemiological Studies Depression Scale participants scored an average of 11.17 (SD = 4.08). On this scale, ranging from 0 to 60, higher scores represent higher levels of depression symptoms and Henry et al. (2018) suggested a clinical cutoff of 21 or higher represents depression.

Spatial Memory and Navigation Correlation Tables

	Test 1					Test 2				
		Path Length	Header Direction	Initial Orientation	Spatial Memory		Path Length	Header Direction	Initial Orientation	Spatial Memory
Sleep	Path Length	1				Path Length	1			
	Header Direction	.34	1			Header Direction	.0.145	1		
	Initial Orientation	.176	.055	1		Initial Orientation	-0.076	.432*	1	
	Spatial Memory	-.217	.35	-.089	1	Spatial Memory	-0.342	.384*	.166	1
		Path Length	Header Direction	Initial Orientation	Spatial Memory		Path Length	Header Direction	Initial Orientation	Spatial Memory
Wake	Path Length	1				Path Length	1			
	Header Direction	.511**	1			Header Direction	.491**	1		
	Initial Orientation	0.099	.447*	1		Initial Orientation	0.196	.662**	1	
	Spatial Memory	-0.135	0.343	0.15	1	Spatial Memory	0.069	.436*	0.233	1

Table S1. Testing session correlations within testing sessions at the individual level between spatial memory and navigation metrics. Asterisks are the following: $p < .001$**, $p < .05$*.

	Test 1					Test 2				
		Path Length	Header Direction	Initial Orientation	Spatial Memory		Path Length	Header Direction	Initial Orientation	Spatial Memory
Sleep	Path Length	1				Path Length	1			
	Header Direction	.260**	1			Header Direction	.293**	1		
	Initial Orientation	.123*	.576**	1		Initial Orientation	.232**	.633**	1	
	Spatial Memory	0.03	.220**	0.088	1	Spatial Memory	0.043	.198**	0.097	1
		Path Length	Header Direction	Initial Orientation	Spatial Memory		Path Length	Header Direction	Initial Orientation	Spatial Memory
Wake	Path Length	1				Path Length	1			
	Header Direction	.269**	1			Header Direction	.288**	1		
	Initial Orientation	0.104	.562**	1		Initial Orientation	.148**	.599**	1	
	Spatial Memory	-0.008	0.072	0.027	1	Spatial Memory	0.019	.230**	.168**	1

Table S2. Testing session correlations within testing sessions at the object-by-object level between spatial memory and navigation metrics. Asterisks are the following: $p < .001$**, $p < .05$*.
